# Metacognitive discrepancies in schizotypy: Divergence between subjective and objective cognitive functioning

**DOI:** 10.1016/j.scog.2026.100428

**Published:** 2026-03-18

**Authors:** Parth Nakirikanti, Jeffrey E. Cassisi, Jeffrey S. Bedwell

**Affiliations:** a College of Sciences, Department of Psychology, University of Central Florida, United States of America; b College of Medicine, Burnett School of Biomedical Sciences, University of Central Florida, United States of America

**Keywords:** Schizotypy, Metacognition, Subjective–objective discrepancy, sustained attention, Working memory

## Abstract

Metacognitive discrepancies between subjective and objective cognition are increasingly recognized across the schizophrenia spectrum but remain understudied in nonclinical schizotypy. This study examined how three schizotypy dimensions (Cognitive-Perceptual, Interpersonal, and Disorganized) relate to divergence between perceived and measured cognitive functioning in emerging adults. In a large online sample of undergraduate students (*N* = 1039), participants completed self-report measures of schizotypy and perceived cognitive functioning (WHODAS 2.0 Cognition domain) alongside laboratory-style tasks of sustained attention (Continuous Performance Test: Identical Pairs, CPT-IP) and visuospatial working memory (2-back and 3-back). Subjective and objective scores were standardized and combined into a Subjective-Objective Discrepancy Index (SODI), with more negative values reflecting greater perceived cognitive difficulty than indicated by task performance. All three schizotypy domains were significantly associated with greater perceived cognitive difficulty relative to objective performance. Individuals higher in schizotypy reported more cognitive impairment than was reflected in their task performance. Consistent with this pattern, higher scores on all three domains predicted lower self-reported cognitive functioning. In contrast, objective cognitive performance showed a dimension-specific profile: Cognitive-Perceptual schizotypy was associated with lower performance on the combined sustained attention and working memory index, Interpersonal schizotypy showed no significant association, and Disorganized schizotypy demonstrated a small, unexpected positive association with objective performance. These findings indicate consistent metacognitive discrepancies in schizotypy, support the SODI as an integrative index of subjective–objective divergence, and suggest that metacognitive processes may represent an important consideration when conceptualizing cognition and functioning across the psychosis spectrum.

## 1 Introduction

Schizotypy is characterized by traits that mirror subclinical expressions of schizophrenia-spectrum disorders and that are closely linked to cognitive and metacognitive functioning (Barrantes-Vidal et al., 2013; Kwapil et al., 2013; Claridge and Davis, 2003; Ettinger et al., 2015). These traits are commonly organized into three empirically derived dimensions: Cognitive-Perceptual (positive traits such as ideas of reference and unusual experiences), Interpersonal (negative traits such as restricted affect and social withdrawal), and Disorganized (disorganized thinking and speech) (Barrantes-Vidal et al., 2013; Kwapil et al., 2013; Claridge and Davis, 2003). These dimensions parallel the positive, negative, and disorganized symptom clusters of schizophrenia, and are typically assessed psychometrically using self-report questionnaires. Dimensional approaches to schizotypy preserve individual variability that is obscured by categorical distinctions, which is especially valuable for studying cognition and its correlates across the schizophrenia spectrum in nonclinical samples.

One area of growing interest involves differences between subjective perceptions and objective indicators of cognitive functioning, often referred to as metacognitive discrepancies (Nelson, 1990; Mitchell and Cohen, 2017; Rouy et al., 2021). Metacognition encompasses awareness and regulation of one's own cognitive processes, including monitoring performance and updating self-beliefs about cognitive ability (Nelson, 1990; Mitchell and Cohen, 2017; Rouy et al., 2021; Davies and Greenwood, 2020; Myers et al., 2024). Discrepancies in which individuals' self-assessments diverge from measured performance have been documented in schizophrenia and in nonclinical schizotypy samples (Chan et al., 2015; Chun et al., 2013; Cohen et al., 2014; Lehmann and Ettinger, 2023; Davies and Greenwood, 2020; Abbasi Sarajehlou et al., 2023). In schizophrenia, metacognitive discrepancies are well established and are associated with worse functional outcomes, including poorer community functioning, reduced social participation, and lower quality of life (Davies and Greenwood, 2020; Arnon-Ribenfeld et al., 2017; Myers et al., 2024; Rouy et al., 2021). Emerging evidence suggests qualitatively similar, although more subtle, patterns in schizotypy: individuals high in schizotypy often report marked cognitive difficulties despite little or no objective impairment on standardized tasks, and they endorse lower quality of life even when objective indicators do not differ (Chan et al., 2015; Chun et al., 2013; Cohen et al., 2014; Lehmann and Ettinger, 2023). This “subjective–objective paradox” suggests that self-assessments of cognitive functioning in schizotypy may be shaped by negative cognitive biases, limited self-monitoring, and reduced insight, and that these metacognitive distortions may be important targets for intervention across the psychosis spectrum.

Alongside these subjective–objective discrepancies, schizotypy has also been linked to reduced objective neurocognitive performance, particularly in sustained attention and working memory, which are central domains of schizophrenia-related cognitive impairment (Ettinger et al., 2015; Abbasi Sarajehlou et al., 2023; Gooding et al., 2006; Meller et al., 2019). Sustained attention, defined as the ability to maintain focus over time, is often reduced among individuals with elevated schizotypy, especially within the Cognitive-Perceptual and Interpersonal dimensions (Abbasi Sarajehlou et al., 2023; Gooding et al., 2006; Meller et al., 2019). In contrast, working memory findings show a more nuanced pattern across schizotypy dimensions and task demands. Working memory, the temporary storage and manipulation of information, appears relatively intact on simple maintenance tasks but shows decrements on higher-load tasks that require both maintenance and manipulation, such as the visuospatial n-back, particularly among individuals high in Cognitive-Perceptual schizotypy (Park et al., 1995; Schmidt-Hansen and Honey, 2009; Kerns and Becker, 2008; Siddi et al., 2017; Abbasi Sarajehlou et al., 2023). Interpersonal and Disorganized schizotypy have shown less consistent associations with working memory and broader executive functioning, suggesting that cognitive deficits may be domain- and dimension-specific (Park et al., 1995; Schmidt-Hansen and Honey, 2009; Kerns and Becker, 2008; Siddi et al., 2017; Abbasi Sarajehlou et al., 2023; Kane et al., 2016; Moat et al., 2024).

Despite converging evidence for both subjective–objective discrepancies and domain-specific neurocognitive inefficiencies, few studies have integrated objective cognitive performance and self-reported judgments of cognitive ability within a single analytic framework (Ettinger et al., 2015; Mitchell and Cohen, 2017; Chan et al., 2015; Chun et al., 2013; Cohen et al., 2014; Lehmann and Ettinger, 2023; Park et al., 1995; Schmidt-Hansen and Honey, 2009; Garg et al., 2025). Many prior schizotypy studies have dichotomized participants into “high” and “low” groups, reducing sensitivity to dimensional variation and limiting the precision with which subjective–objective mismatches can be quantified. Recent work suggests that schizotypy dimensions may be associated with alterations in metacognitive monitoring and introspective accuracy, including reduced correspondence between subjective self-assessments and objective performance, altered confidence calibration, and dysfunctional metacognitive beliefs about cognition (e.g., 11, 16–17). Although the specific cognitive correlates of Cognitive-Perceptual, Interpersonal, and Disorganized traits may differ, distortions in how individuals evaluate and interpret their own cognitive functioning may represent a broader metacognitive feature of schizotypy. Critically, it appears that no published study has simultaneously examined sustained attention, working memory, and perceived cognitive functioning using matched subjective and objective measures in the same sample of emerging adults, nor has any work applied a standardized discrepancy index that directly captures divergence between perceived and measured cognitive functioning in schizotypy (Ettinger et al., 2015; Mitchell and Cohen, 2017; Chan et al., 2015; Chun et al., 2013; Cohen et al., 2014; Lehmann and Ettinger, 2023; Davies and Greenwood, 2020; Myers et al., 2024; Park et al., 1995; Schmidt-Hansen and Honey, 2009; Garg et al., 2025).

Understanding subjective–objective discrepancies in schizotypy has important implications. If individuals with elevated schizotypal traits systematically underestimate their cognitive abilities, this may influence academic functioning, help-seeking behavior, and self-efficacy even in the absence of objective impairment. Conversely, if discrepancies reflect early alterations in introspective accuracy, they may represent a potential mechanism linking subclinical traits to later functional outcomes. Clarifying whether discrepancies reflect global cognitive impairment or biased self-appraisal is therefore critical for both theoretical models of psychosis-spectrum vulnerability and early identification strategies.

The present study addresses these gaps by investigating how multidimensional schizotypy relates to both objective cognitive performance (sustained attention and working memory) and perceived cognitive functioning in a large nonclinical sample of emerging adults, using a standardized Subjective–Objective Discrepancy Index (SODI) to operationalize metacognitive discrepancy (Mitchell and Cohen, 2017; Chan et al., 2015; Chun et al., 2013; Cohen et al., 2014; Lehmann and Ettinger, 2023; Davies and Greenwood, 2020; Myers et al., 2024; Garg et al., 2025; Cornblatt et al., 1988; Kirchner, 1958; Backx et al., 2020). Objective cognition was assessed using validated measures of sustained attention and working memory (Cornblatt et al., 1988; Kirchner, 1958; Backx et al., 2020), and perceived cognitive functioning was indexed using the Cognition domain of the World Health Organization Disability Assessment Schedule 2.0 (WHODAS 2.0), a measure of self-reported cognitive difficulty in everyday life (Üstün et al., 2010). By modeling the Cognitive-Perceptual, Interpersonal, and Disorganized dimensions simultaneously and relating them to subjective cognition, objective cognition, and their discrepancy, this study provides a direct test of the subjective–objective paradox in schizotypy and clarifies how alterations in introspective accuracy interface with core cognitive domains central to schizophrenia research (Ettinger et al., 2015; Mitchell and Cohen, 2017; Chan et al., 2015; Chun et al., 2013; Cohen et al., 2014; Lehmann and Ettinger, 2023; Davies and Greenwood, 2020; Myers et al., 2024; Park et al., 1995; Schmidt-Hansen and Honey, 2009; Garg et al., 2025; Cornblatt et al., 1988; Kirchner, 1958; Backx et al., 2020).

The SODI is conceptually aligned with the construct of introspective accuracy (IA), defined as the degree to which individuals' self-assessments correspond to objective cognitive performance (Silberstein and Harvey, 2019; Silberstein et al., 2018). IA has been extensively studied in schizophrenia-spectrum disorders, where reduced correspondence between perceived and measured cognition has been linked to functional outcomes. The SODI operationalizes this correspondence as a standardized difference score capturing both the magnitude and direction of divergence between subjective and objective cognition. Thus, rather than introducing a distinct construct, the present study extends IA research by examining subjective–objective divergence within a multidimensional schizotypy framework in a large nonclinical sample.

Although objective neurocognitive findings in schizotypy appear to vary by dimension, metacognitive distortions may reflect a more general process that cuts across positive-like, negative-like, and disorganized traits. Each schizotypy dimension has been linked to cognitive biases or altered self-referential processing that could plausibly influence how individuals evaluate their own cognitive functioning. For example, Cognitive-Perceptual traits involve unusual beliefs and perceptual experiences that may foster mistrust in one's own mental processes; Interpersonal traits are characterized by social withdrawal and negative self-appraisal, which may generalize to perceptions of cognitive competence; and Disorganized traits involve thought disorganization that may reduce confidence in one's cognitive clarity even in the absence of measurable impairment. Thus, while objective cognitive deficits may be dimension-specific, distortions in self-appraisal may operate more broadly across schizotypy dimensions. On this basis, we hypothesized that all three schizotypy dimensions would be associated with greater subjective–objective discrepancy.

### 1.1 Hypotheses

H1 **Subjective-Objective Discrepancy**

Higher scores on the Cognitive-Perceptual, Interpersonal, and Disorganized schizotypy domains will be independently associated with more negative values on the Subjective-Objective Discrepancy Index (SODI), indicating that individuals perceive greater cognitive difficulties than are evident in their objective task performance.

H2 **Subjective Cognitive Functioning**

Higher scores on all three schizotypy domains will be associated with lower levels of self-reported cognitive ability (lower reversed WHODAS Cognition scores), reflecting reduced self-perceived subjective cognitive function.

H3 **Objective Cognitive Performance**

Higher scores in the Cognitive-Perceptual schizotypy domain will be associated with lower objective cognitive performance, reflected in lower averaged standardized scores across the CPT-IP and n-back tasks, whereas associations involving the Interpersonal and Disorganized domains are examined exploratorily given mixed findings in the literature.

## 2 Method

### 2.1 Participants

From January to September 2023, undergraduate students enrolled in psychology courses at a large United States public university were recruited via an online participant pool for course credit. The institutional review board (IRB) reviewed and approved the study protocol as exempt from full review because data were collected and stored anonymously with no linked personal identifiers. This determination was formally documented by the IRB; the protocol was not categorized based solely on investigator determination of exempt status. Of the 1698 students who initiated the study, exclusions were made based on the following a priori criteria: 40 participants were excluded for not completing the schizotypy questionnaire; 424 participants were excluded for exceeding the Attentive Responding Scale (ARS) cutoff (scores ≥11.5) indicating inattentive responding; and 195 participants were excluded for failing to complete both neurocognitive tasks. The final sample included 1039 participants (mean age = 19.4 years, SD = 1.74, range = 18 to 28; 59% women; 51% White, 19% Hispanic/Latinx, 12% Asian, 10% Black/African American, 8% Multiracial or Other). Demographic characteristics did not significantly differ between those included and excluded from the final sample on age or sex (*p*s > 0.05).

The study did not include formal screening for prior psychiatric diagnoses, and participants were not excluded based on self-reported mental health history. As the sample was drawn from a nonclinical undergraduate population and participation was anonymous, diagnostic status was not assessed. Thus, the present findings reflect schizotypy and cognitive functioning in a naturalistic emerging adult sample rather than a diagnostically characterized cohort.

### 2.2 Measures

#### 2.2.1 Schizotypal Personality Questionnaire-Brief Revised (SPQ-BR)

The SPQ-BR (Callaway et al., 2014) is a widely used 32-item self-report measure of schizotypy traits derived from DSM-III-R criteria for schizotypal personality disorder (Raine, 1991). Items are rated on a 5-point Likert scale from 1 (strongly disagree) to 5 (strongly agree). The questionnaire yields three subscale scores: Cognitive-Perceptual (odd beliefs, unusual perceptions, ideas of reference), Interpersonal (social anxiety, no close friends, constricted affect), and Disorganized (eccentric behavior, odd speech). In the current sample, internal consistency was high: Cognitive-Perceptual (Cronbach's alpha, α = 0.87), Interpersonal (α = 0.85), Disorganized (α = 0.81). These values are consistent with published estimates from prior studies (α = 0.83–0.93) (Callaway et al., 2014). Subscale scores were analyzed separately as continuous variables to preserve dimensional variation in schizotypy.

#### 2.2.2 World Health Organization Disability Assessment Schedule 2.0 (WHODAS)

The WHODAS (Üstün et al., 2010) is a 36-item self-report instrument assessing functional ability across six domains: cognition, mobility, self-care, getting along, life activities, and participation. The present study focused exclusively on the Cognition domain, consisting of 6 items (e.g., “In the last 30 days, how much difficulty did you have concentrating on doing something for ten minutes?”). Items are rated on a 5-point scale from 1 (none) to 5 (extreme), and a standard scoring algorithm is applied. Raw domain scores are converted to a 0–100 scale using WHO guidelines, with higher values reflecting greater perceived disability. The WHODAS Cognition domain evaluates perceived difficulties in memory, concentration, learning, and comprehension in everyday life over the past 30 days. Because items are anchored to real-world task demands rather than structured laboratory performance, the WHODAS Cognition domain captures subjective cognitive functioning as experienced in daily contexts (Üstün et al., 2010)Internal consistency for the Cognition domain in the current sample was excellent (α = 0.94), consistent with published estimates (Üstün et al., 2010).

#### 2.2.3 Attentive Responding Scale (ARS)

The infrequency subscale of the ARS (Maniaci and Rogge, 2014) is an 11-item validity scale designed to detect inattentive or careless responding to self-report questionnaires. Items include statements such as “I don't like getting speeding tickets” and “My favorite subject is agronomy,” and are rated on a 5-point Likert scale from 1 (strongly disagree) to 5 (strongly agree). A cutoff score of ≥11.5 (i.e., sum of raw item responses) identifies participants whose response pattern suggests inadequate attention to items and is associated with reduced data quality in psychological research (Maniaci and Rogge, 2014). This threshold was empirically derived and validated in a large sample (Maniaci and Rogge, 2014). In the current sample, internal consistency was high (α = 0.83). The ARS was embedded among self-report questionnaire items in random order to minimize demand characteristics.

#### 2.2.4 Continuous Performance Test-Identical Pairs (CPT-IP)

The CPT-IP (Cornblatt et al., 1988) is a computerized measure of sustained attention administered online through Pavlovia. Participants viewed 150 four-digit numbers presented sequentially in the center of the screen (presentation duration = 50 ms each, interstimulus interval = 2 s). Participants were instructed to respond by pressing the spacebar when a stimulus matched the immediately preceding number (target trials). Thirty practice trials preceded 150 experimental trials. Experimental trials included a random presentation of 30 target trials (in which the current number matched the prior number) and 28 false-alarm trials (trials in which the number presented differed in only one digit from the previous; the remaining 92 trials were non-target, non-false-alarm trials). The primary outcome was d' (discriminability), a signal detection measure calculated as Z(hit rate) – Z(false alarm rate), where hit rate represents the proportion of correct responses to target trials and false alarm rate represents the proportion of incorrect responses to non-target trials. Higher d′ values reflect greater sensitivity in discriminating target from non-target stimuli, which in the CPT-IP is interpreted as better sustained attention performance (Cornblatt et al., 1988; Macmillan and Creelman, 2005)

#### 2.2.5 Visuospatial n-back task

The visuospatial n-back task (Kirchner, 1958) measures working memory under varying cognitive load, administered online through Pavlovia using PsychoPy alongside the CPT-IP (Peirce et al., 2019). On each trial, a white square appeared in one of eight locations arranged in a circle around the screen center. Participants pressed the spacebar if the current stimulus matched the stimulus presented *n* trials earlier. The task progressed through three conditions (1-back, 2-back, and 3-back), where *n* = the number of trials back. Each condition contained 120 trials with 25% target trials (i.e., 30 target trials per condition), with the remaining trials as non-targets. Stimuli were presented for 500 ms with a 2500 ms interstimulus interval. Analyses focused on the 2-back and 3-back conditions, which impose substantial working memory demands. Performance on each condition was indexed by d' (discriminability), calculated identically to the CPT-IP using hit rates and false alarm rates.

#### 2.2.6 Procedure

All study procedures were completed online. After providing informed consent through an online participant pool platform, participants were redirected to Qualtrics (www.qualtrics.com) to complete (in order): demographic questions, the SPQ-BR, the WHODAS 2.0 (with ARS items embedded randomly throughout). Participants then were directed to Pavlovia (www.pavlovia.org) to complete the objective cognitive measures (CPT-IP followed by n-back task) (Peirce et al., 2019). The median completion time for the entire study was approximately 35 min. Participants received course credit as compensation.

### 2.3 Data quality assurance procedures

Because neurocognitive tasks were administered in an online, uncontrolled environment, several procedures were implemented to promote data integrity. First, participants completed the Attentive Responding Scale (ARS) as an embedded validity measure within the self-report battery. A cutoff score of ≥11.5 was used to identify inattentive or careless responding, consistent with established guidelines (Maniaci and Rogge, 2014). Participants exceeding this threshold were excluded from all analyses.

Second, trial-level response time and accuracy data from the cognitive tasks were examined to identify patterns suggestive of disengagement (e.g., extended sequences of extremely rapid responses or nonresponses). Although no additional trial-level exclusions were implemented beyond the ARS criterion, performance distributions were inspected to ensure consistency with engaged task completion.

Third, cognitive tasks were administered using established experimental software in a web-based format. Prior research has demonstrated acceptable correspondence between web-based and laboratory-based administration of cognitive tasks, particularly for signal detection paradigms (Cornblatt et al., 1988; Backx et al., 2020; Peirce et al., 2019). These procedures were implemented to balance feasibility and ecological validity with data quality safeguards in an online testing context.

### 2.4 Statistical analyses

#### 2.4.1 Variable construction

**Subjective Cognitive Performance.** The WHODAS Cognition domain scores were standardized (z-scores) such that higher values indicated better self-perceived cognitive functioning (i.e., reverse-coded and standardized).
**Objective Cognitive Performance.** Objective cognitive performance was calculated in two steps. First, d' scores from the 2- and 3-back tasks were standardized (z-scores) and averaged to create a composite n-back score. This composite n-back d' score was then standardized and averaged with the standardized d' score from the CPT-IP to yield a composite index of signal detection sensitivity across both tasks. Higher values reflect greater accuracy and efficiency in cognitive processing across sustained attention and working memory domains. This composite approach was used to create a unified objective cognition construct comparable to the broad WHODAS Cognition domain.
  The use of a composite objective cognition score is consistent with common approaches in schizophrenia-spectrum research, in which standardized performance across multiple neurocognitive tasks is aggregated to provide a more stable estimate of general cognitive functioning and to reduce task-specific measurement error. Composite indices are frequently employed when examining associations between cognition and clinical or functional outcomes, as they capture shared variance across tasks while minimizing idiosyncratic features of any single paradigm. In the present study, combining sustained attention (CPT-IP) and working memory (n-back) performance yielded a broader objective cognition construct that more closely parallels the WHODAS Cognition domain, which reflects global perceived cognitive functioning rather than performance in a single laboratory task. This approach therefore enhances conceptual comparability between subjective and objective cognition while maintaining sensitivity to core cognitive domains implicated in schizotypy.
**Subjective-Objective Discrepancy Index (SODI).** The SODI was calculated by subtracting standardized objective performance scores from standardized subjective cognition scores (SODI = subjective – objective). Larger negative values on the SODI indicate that participants perceived more cognitive difficulties (lower subjective scores) than were evident in objective task performance (higher objective scores), reflecting subjective underestimation of cognitive ability. Larger positive values indicate subjective overestimation. The SODI provides a standardized, interpretable metric of the magnitude and direction of the subjective-objective discrepancy.

#### 2.4.2 Analytic strategy

Three separate linear regression models tested associations between schizotypy domain scores (SPQ-BR Cognitive-Perceptual, Interpersonal, and Disorganized subscales entered simultaneously as predictors) and three outcomes: (a) SODI (H1), (b) subjective cognition (H2), and (c) objective cognition (H3). The SODI model included objective cognition as a covariate to account for individual differences in objective performance, which influences the subtraction score. Sex was included as a covariate in all three models because independent-samples *t*-tests revealed statistically significant differences between men and women on both objective cognition, t(1037) = 3.42, *p* < .001 (men: M = 0.19, SD = 0.95; women: M = −0.11, SD = 1.01), and subjective cognition, t(1037) = 4.15, p < .001 (men: M = −0.19, SD = 0.98; women: M = 0.12, SD = 1.00). Unstandardized regression coefficients (B) and standardized coefficients (β) are reported. Model fit was evaluated using adjusted R^2^ and variance inflation factors (VIF) to assess multicollinearity. All analyses were conducted in SPSS version 30.0.

## 3 Results

### 3.1 Descriptive statistics

Table 1 presents means and standard deviations for the study variables. Means and standard deviations for the SPQ-BR subscales were comparable to values reported in previous studies of nonclinical samples (Barrantes-Vidal et al., 2013; Callaway et al., 2014). The mean SODI was negative (M = −0.11, SD = 0.95), suggesting that on average, participants reported slightly more cognitive difficulties than their objective performance would predict.

**Table 1:** Primary study measures by biological sex.

Variable	Male M (SD)	Female M (SD)	Total M (SD)	F(df₁, df₂), p
**SPQ-BR**				
Cognitive-Perceptual	33.86 (8.26)	38.63 (8.94)	36.69 (8.98)	75.82 (1,1034)**
Interpersonal	28.22 (7.99)	30.42 (8.13)	29.52 (8.14)	18.50 (1,1034)**
Disorganized	25.31 (6.41)	26.62 (6.16)	26.09 (6.29)	11.09 (1,1034)**
**WHODAS**				
Cognition (Do1)	19.95 (15.70)	24.93 (16.89)	22.91 (16.59)	22.94 (1,1034)**
**CPT-IP**				
Hits	24.47 (6.22)	23.89 (5.41)	24.13 (5.76)	2.54 (1,1034), N.S.
False alarms	6.94 (7.68)	9.78 (12.89)	8.63 (11.16)	16.38 (1,1034)**
Misses	5.49 (6.13)	6.04 (5.32)	5.82 (5.67)	2.42 (1,1034), N.S.
d′	0.21 (1.41)	−0.14 (1.64)	0.00 (1.56)	13.01 (1,1034)**
**Visuospatial n-back**				
2-Back hits	10.06 (3.99)	9.04 (3.77)	9.45 (3.89)	17.37 (1,1034)**
2-Back false alarms	2.91 (4.79)	3.72 (5.54)	3.39 (5.26)	5.85 (1,1034)*
2-Back misses	3.86 (3.93)	4.88 (3.72)	4.47 (3.84)	18.07 (1,1034)**
3-Back hits	7.13 (3.83)	5.81 (3.24)	6.35 (3.55)	35.71 (1,1034)**
3-Back false alarms	5.00 (5.93)	5.26 (6.39)	5.15 (6.21)	0.42 (1,1034), N.S.
3-Back misses	7.76 (3.84)	9.04 (3.31)	8.52 (3.59)	32.41 (1,1034)**
d′ (average)	0.25 (1.32)	−0.17 (1.22)	0.00 (1.28)	26.84 (1,1034)**

Note. F statistics and *p*-values are from one-way ANOVAs comparing males and females. SPQ-BR = Schizotypal Personality Questionnaire–Brief Revised (Silberstein et al., 2018). WHODAS Cognition = World Health Organization Disability Assessment Schedule 2.0 Cognition Domain (raw scores on 0–100 scale, higher values indicate greater perceived cognitive disability) (Backx et al., 2020). CPT-IP = Continuous Performance Test–Identical Pairs (Cornblatt et al., 1988). Visuospatial n-Back (Kirchner, 1958).

N.S. = not significant; *p* < .05 (*); *p* < .01 (**), two-tailed.

Table 2 provides zero-order correlations among the study variables and the three schizotypy domain scores. The schizotypy domain scores were significantly negatively correlated with subjective cognition (r's ranging from −0.35 to −0.46), indicating that higher schizotypy levels were associated with worse self-reported cognitive functioning. Correlations between schizotypy domain scores and objective cognition were weaker and more variable (rs ranging from −0.12 to 0.07), consistent with previous literature. The SODI was significantly negatively correlated with all three schizotypy dimensions (rs ranging from −0.20 to −0.40), indicating that higher schizotypy was associated with larger negative discrepancies (i.e., underestimation of ability relative to objective performance).

**Table 2:** Zero-order correlations among study variables.

Variable	1	2	3	4	5	6	7
1. Biological sex	–						
2. Cognitive perceptual schizotypy	0.26**	–					
3. Interpersonal schizotypy	0.13**	0.37**	–				
4. Disorganized schizotypy	0.10**	0.42**	0.33**	–			
5. Subjective cognitive functioning	−0.15**	−0.35**	−0.37**	−0.46**	–		
6. Objective cognitive functioning	−0.16**	−0.12**	0.01	0.07*	−0.00	–	
7. SODI	−0.01	−0.20**	−0.29**	−0.40**	0.77**	−0.64**	–

Note. *N* = 1036. Biological sex = participant self-reported biological sex (0 = male, 1 = female). Cognitive-Perceptual, Interpersonal, and Disorganized schizotypy were measured with the Schizotypal Personality Questionnaire–Brief Revised (SPQ-BR). Subjective cognitive functioning = standardized reversed WHODAS 2.0 Cognition domain scores, with higher values indicating better perceived cognitive functioning. Objective cognitive functioning = standardized composite of CPT-IP and visuospatial n-back d' performance, with higher values reflecting better objective cognitive performance. SODI = Subjective–Objective Discrepancy Index, calculated as Subjective Cognitive Score minus Objective Cognitive Score; more negative SODI values indicate greater perceived cognitive difficulties than are evident in objective performance.

p < .05 (*), p < .01 (**), two-tailed.

### 3.2 Subjective-Objective Discrepancy Index (SODI)

A linear regression model examined the SODI as the outcome, predicted by the three schizotypy domains (Cognitive-Perceptual, Interpersonal, Disorganized), with sex and objective cognition included as covariates. The overall model was significant, F(5, 1033) = 282.53, *p* < .001, adjusted R^2^ = 0.58. Variance inflation factors were all below 1.45, indicating acceptable multicollinearity. Each schizotypy domain was significantly and negatively associated with the SODI. Specifically, the Cognitive-Perceptual domain showed a negative association (β = −0.10, *p* < .001), the Interpersonal domain demonstrated a stronger negative association (β = −0.16, p < .001), and the Disorganized domain exhibited the strongest negative association (β = −0.26, p < .001).

**Fig. 1:**
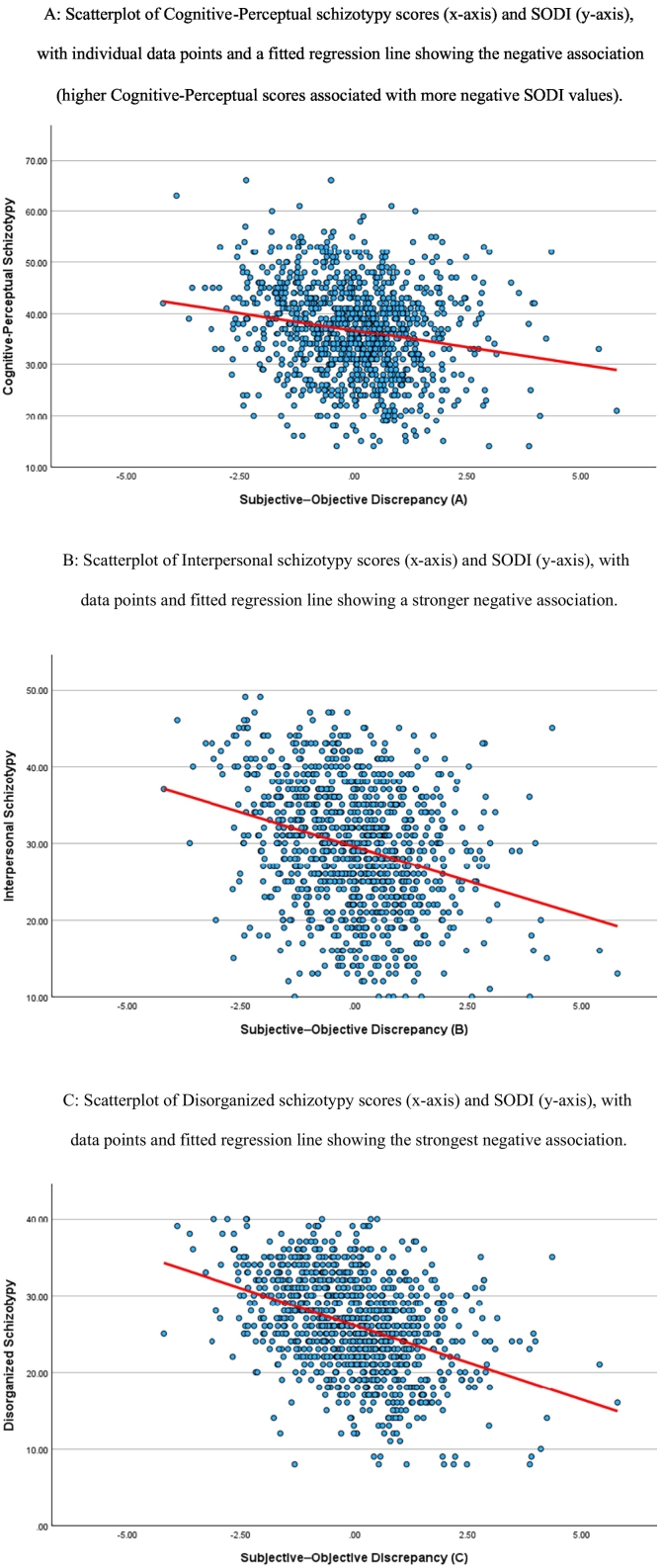
Relationships between schizotypy dimensions and the Subjective–Objective Discrepancy Index (SODI). Scatterplots illustrate associations between SODI and (A) Cognitive-Perceptual, (B) Interpersonal, and (C) Disorganized subscales of the SPQ-BR, with fitted regression lines. SODI was calculated as Subjective Cognitive Score minus Objective Cognitive Score; more negative values indicate that participants perceived greater cognitive difficulties than were evident in objective task performance.

The negative associations indicate that higher schizotypy across all three domains was associated with more negative SODI values, meaning that individuals with elevated schizotypy perceived greater cognitive difficulties relative to their objective task performance. Standardized regression coefficients ranged from β = −0.10 (Cognitive-Perceptual) to β = −0.26 (Disorganized), reflecting statistically reliable but incrementally sized associations within the multivariate model. Although the overall model accounted for a substantial proportion of variance in the SODI (adjusted R^2^ = 0.58), the unique contributions of individual schizotypy dimensions were modest in magnitude, consistent with multifactorial influences on subjective–objective discrepancies. Scatterplots illustrating these relationships are presented in Fig. 1.

### 3.3 Subjective cognitive functioning

A linear regression model predicting subjective cognition from the three schizotypy domains and sex was also significant, F(4, 1034) = 101.86, *p* < .001, adjusted R^2^ = 0.28, VIF < 1.40. All three schizotypy domains were negatively related to subjective cognition. The Cognitive-Perceptual domain was associated with lower subjective cognitive functioning (β = −0.12, p < .001), the Interpersonal domain showed a stronger negative relationship (β = −0.20, p < .001), and the Disorganized domain demonstrated the strongest negative association (β = −0.34, p < .001).

These negative associations indicate that higher schizotypy across all dimensions was associated with higher self-reported cognitive disability (lower standardized subjective cognition scores), reflecting worse perceived cognitive functioning. Again, effect sizes were small to moderate, with Disorganized schizotypy showing the strongest relationship. Scatterplots are presented in Fig. S1 (Supplementary materials).

### 3.4 Objective cognitive performance

A linear regression model examined associations between schizotypy domains and objective cognitive performance, controlling for sex. The overall model was significant, F(4, 1034) = 14.06, *p* < .001, adjusted R^2^ = 0.05, VIF < 1.40. Results revealed differential patterns across schizotypy dimensions. The Cognitive-Perceptual domain was significantly associated with lower performance (β = −0.15, p < .001), the Interpersonal domain showed no significant association (β = 0.04, *p* = .28), and the Disorganized domain was linked to better performance (β = 0.14, p < .001).

In support of H3, higher Cognitive-Perceptual schizotypy was associated with lower objective cognitive performance. Contrary to H3, Interpersonal schizotypy showed no significant association with objective performance. Unexpectedly, higher Disorganized schizotypy was associated with better objective cognitive performance, a result that warrants discussion. Scatterplots are presented in Fig. S2 (Supplementary materials).

## 4 Discussion

### 4.1 Summary of findings

This study examined how multidimensional schizotypy relates to perceived cognitive functioning, objective cognitive performance, and their divergence in a large undergraduate sample (*N* = 1039). Metacognitive discrepancy was indexed by the Subjective–Objective Discrepancy Index (SODI), a standardized difference score reflecting the extent to which the Subjective Cognitive Score (reversed and standardized WHODAS Cognition) diverges from the Objective Cognitive Score (composite CPT-IP and visuospatial n-back d').

Consistent with H1, H2, higher Cognitive-Perceptual, Interpersonal, and Disorganized schizotypy were associated with more negative SODI values and with lower Subjective Cognitive Scores, indicating that individuals with elevated schizotypy tend to report greater cognitive difficulties than are evident in their performance and endorse worse perceived cognition in daily life. Objective cognitive performance showed a domain-specific pattern partially consistent with H3: higher Cognitive-Perceptual schizotypy predicted lower Objective Cognitive Scores, Interpersonal schizotypy showed no association, and Disorganized schizotypy showed a small positive association (β = 0.14, ∼2% of the variance) that warrants cautious interpretation and replication.

### 4.2 Interpretation of key findings

#### 4.2.1 Subjective-objective discrepancies

The associations between all three schizotypy domains and the SODI constitute a central finding. The SODI provides a standardized quantitative index of divergence between self-reported and task-based cognitive functioning, capturing the extent to which individuals underestimate or overestimate their abilities relative to measured performance; more negative values indicate underestimation of cognitive ability. In this sample, higher Cognitive-Perceptual, Interpersonal, and Disorganized scores were each linked to more negative SODI values, with the largest effect for Disorganized schizotypy, indicating that metacognitive discrepancies are evident across positive-like, negative-like, and disorganization-related traits. This pattern extends previous research documenting mismatches between subjective complaints and objective performance in psychosis-spectrum conditions (Chan et al., 2015; Cohen et al., 2014; Lehmann and Ettinger, 2023) and is consistent with the “subjective–objective paradox,” in which individuals report substantial cognitive difficulty despite relatively intact task performance (Cohen et al., 2014).The present findings also align with earlier studies on IA in schizophrenia-spectrum conditions. IA research has demonstrated that discrepancies between self-reported and objective cognitive functioning are common and clinically meaningful (Silberstein and Harvey, 2019; Silberstein et al., 2018). By using the SODI as a standardized discrepancy metric, the current study extends this literature to multidimensional schizotypy in a nonclinical sample, suggesting that alterations in introspective accuracy may be evident even at subclinical levels of psychosis-spectrum traits.

The WHODAS Cognition domain, by itself, assesses perceived difficulties with memory, concentration, learning, and comprehension in everyday life and does not directly measure metacognition. Metacognitive inferences in this study arise from the discrepancy between this subjective index and objective task performance, as captured by the SODI, rather than from a dedicated metacognition scale. For example, validated self-report instruments such as the Metacognitive Awareness Inventory (Schraw and Dennison, 1994) or the Beck Cognitive Insight Scale (Beck et al., 2004) can assess general self-reflectiveness and awareness of cognitive processes. The SODI should therefore be viewed as an index of subjective–objective divergence that is compatible with, but not equivalent to, direct assessments of metacognitive monitoring and insight. Future research using explicit metacognitive measures (for example, confidence ratings, metacognitive questionnaires, or trial-by-trial monitoring paradigms) will be important for clarifying whether SODI-related discrepancies are driven by biased self-beliefs, limited performance monitoring, or other metacognitive processes.

Importantly, the presence of subjective–objective discrepancies across schizotypy dimensions suggests that metacognitive distortion may operate partly independently of measurable neurocognitive impairment. In the present study, objective performance deficits were dimension-specific, whereas divergence between perceived and measured cognition was observed across Cognitive-Perceptual, Interpersonal, and Disorganized traits. This pattern supports the interpretation that biased self-appraisal or altered introspective calibration may represent general characteristic of schizotypy across symptom dimensions, rather than simply a downstream consequence of objective cognitive inefficiency. Such discrepancies may reflect negatively biased beliefs about cognitive competence, heightened sensitivity to everyday cognitive lapses, or reduced confidence in cognitive clarity, even when laboratory performance remains intact. This interpretation is consistent with prior work demonstrating that subjective cognitive complaints in psychosis-spectrum conditions are often only weakly associated with objective performance but are more strongly related to affective distress and self-evaluative processes (Cohen et al., 2014; Davies and Greenwood, 2020).

Conceptually, these findings reinforce the distinction between cognitive capacity and cognitive self-appraisal. Whereas objective tasks index performance under structured conditions, subjective reports reflect individuals' interpretations of their cognitive experiences in daily life. Discrepancies between the two may therefore provide unique insight into how individuals construct beliefs about their cognitive functioning, potentially serving as an early marker of vulnerability within the psychosis spectrum.

#### 4.2.2 Differential patterns across schizotypy domains

Dimension-specific associations with the Objective Cognitive Score represent another key contribution. Cognitive-Perceptual schizotypy was associated with lower objective performance on the combined sustained attention and working memory index, consistent with work linking positive-like schizotypy features (such as unusual perceptual experiences and odd beliefs) to vulnerabilities in attention regulation and information processing. In contrast, Interpersonal schizotypy showed no significant association with objective performance, suggesting that social-affective traits may be less directly related to the specific laboratory-based attention and working memory constructs assessed here or may be more strongly tied to other domains such as social cognition. This pattern is consistent with prior findings suggesting that negative-like or interpersonal schizotypy traits show weaker or more inconsistent associations with performance-based cognitive measures compared to positive-like traits (Siddi et al., 2017; Kane et al., 2016).

The pattern for Disorganized schizotypy was unexpected: higher Disorganized scores were associated with slightly better objective performance, with a small effect size (β = 0.14, approximately 2% of the variance). This finding diverges from some prior work reporting null or negative associations between disorganization and executive or working memory performance (Kerns and Becker, 2008; Siddi et al., 2017). Possible explanations include sampling characteristics (for example, relatively high-functioning students with disorganized traits completing a demanding online protocol), task- or context-specific influences (such as self-paced online administration reducing performance pressure), or the possibility that the selected tasks do not index the aspects of cognition most affected by disorganization (for example, abstraction, cognitive flexibility, or complex reasoning). Given the modest variance explained and potential context effects, replication in independent samples with broader cognitive batteries and varied testing environments will be necessary to determine whether this association reflects a reliable effect or statistical noise.

### 4.3 Methodological strengths and limitations

Several methodological features strengthen the inferences that can be drawn from this study. The large undergraduate sample (*N* = 1039) provided substantial statistical power and allowed schizotypy to be modeled dimensionally rather than via extreme-group designs, preserving graded variability in Cognitive-Perceptual, Interpersonal, and Disorganized traits. The study integrated multiple information sources by combining a validated self-report index of everyday cognitive functioning (WHODAS Cognition), two performance-based neurocognitive tasks (CPT-IP and visuospatial n-back), and a discrepancy metric (SODI) that quantified divergence between subjective and objective cognition within a single analytic framework. The use of established measures with good internal consistency, a composite Objective Cognitive Score that reduced task-specific noise, and rigorous validity screening with the ARS and trial-level inspection further supported data quality in the online context.

Nonetheless, several limitations should be considered. First, the exclusive reliance on undergraduates from a single university limits generalizability to community, clinical, older, or non-U.S. populations. Second, the cross-sectional design precludes causal conclusions about temporal relations among schizotypy, subjective cognition, objective cognition, and their discrepancy; longitudinal or experimental designs will be needed to determine whether schizotypy contributes to metacognitive distortions, whether such distortions influence schizotypy expression, or whether both arise from shared vulnerability factors.

Third, although the WHODAS Cognition domain and the CPT-IP and n-back tasks capture complementary aspects of cognitive functioning, they do not cover the full range of domains implicated in schizophrenia-related impairment. The WHODAS focuses on perceived difficulties with memory, concentration, learning, and comprehension in daily life, whereas the tasks primarily index sustained attention and visuospatial working memory under structured conditions. Broader batteries including verbal learning, processing speed, reasoning, and social cognition would help test whether the observed discrepancies generalize across functions. Fourth, online administration of neurocognitive tasks in uncontrolled environments may have introduced additional variability due to distractions, hardware differences, or internet-related factors; although prior work supports the validity of web-based assessment and multiple data-quality procedures were implemented, residual context effects cannot be ruled out. Finally, the study did not include direct measures of metacognitive monitoring or insight, so inferences about metacognitive processes are based on subjective–objective discrepancies rather than dedicated metacognition instruments; incorporating such measures in future research would clarify mechanisms underlying the subjective–objective paradox in schizotypy.

The absence of diagnostic screening represents a limitation. Although schizotypy is conceptualized dimensionally and studied in nonclinical samples, the inclusion of individuals with unassessed psychiatric conditions may have introduced additional variability. Future work incorporating structured diagnostic assessment would clarify the extent to which the observed subjective–objective discrepancies are independent of clinical diagnoses.

Finally, sex differences were observed in both subjective and objective cognitive indices, with men demonstrating slightly higher objective performance and women reporting slightly better subjective cognitive functioning. Although sex was included as a covariate in all regression models, the present study was not designed to test sex-specific mechanisms underlying subjective–objective discrepancies. Prior work suggests that sex differences in self-evaluative processes and cognitive performance may reflect a combination of sociocultural and cognitive factors, but replication and targeted investigation will be necessary to clarify whether sex moderates the relationship between schizotypy and metacognitive discrepancy.

### 4.4 Clinical and research implications

The consistent association between elevated schizotypy and more negative SODI values indicates that individuals with higher psychosis-spectrum traits often perceive themselves as more cognitively impaired than their objective performance suggests. Clinically, this finding underscores the importance of integrating subjective and performance-based assessments when evaluating cognitive functioning. Reliance solely on subjective complaints may overestimate objective impairment, whereas exclusive reliance on laboratory-based performance may underestimate experienced cognitive difficulty and distress. Incorporating both perspectives can facilitate more accurate case conceptualization and may help distinguish between objective cognitive inefficiency and negatively biased self-appraisal.

From a research perspective, modeling subjective cognition, objective cognition, and their discrepancy simultaneously provides a more nuanced understanding of cognitive functioning across the psychosis spectrum. Longitudinal studies will be necessary to determine whether subjective–objective discrepancies predict functional outcomes, academic performance, help-seeking behavior, or progression along the psychosis continuum. Future research incorporating direct measures of metacognitive monitoring, confidence calibration, and cognitive insight will further clarify the mechanisms underlying subjective–objective divergence and determine whether these discrepancies reflect biased self-beliefs, altered performance monitoring, or other metacognitive processes.

## CRediT authorship contribution statement

**Parth Nakirikanti:** Writing – review & editing, Writing – original draft, Project administration, Methodology, Investigation, Formal analysis, Data curation, Conceptualization. **Jeffrey E. Cassisi:** Writing – original draft, Supervision, Project administration, Methodology, Formal analysis, Data curation, Conceptualization. **Jeffrey S. Bedwell:** Writing – review & editing, Writing – original draft, Methodology, Investigation, Formal analysis, Conceptualization.

## Declaration of competing interest

The authors declare that they have no known competing financial interests or personal relationships that could have appeared to influence the work reported in this paper.

## Appendix A Supplementary data

Fig. S1 Relationships between schizotypy dimensions and the Subjective Cognitive Score. Scatterplots illustrate associations between the Subjective Cognitive Score and (A) Cognitive-Perceptual, (B) Interpersonal, and (C) Disorganized subscales of the SPQ-BR, with fitted regression lines. The Subjective Cognitive Score was calculated by reversing and standardizing the WHODAS 2.0 Cognition domain score; higher values reflect better perceived cognitive functioning, and lower values reflect greater self-reported cognitive difficulties. Fig. S1

Fig. S2 Relationships between schizotypy dimensions and the Objective Cognitive Score. Scatterplots illustrate associations between the Objective Cognitive Score and (A) Cognitive-Perceptual, (B) Interpersonal, and (C) Disorganized subscales of the SPQ-BR, with fitted regression lines. The Objective Cognitive Score was calculated in multiple steps: a d' value was first computed for each participant on the CPT-IP, 2-back, and 3-back tasks; 2-back and 3-back d' values were standardized and averaged to create a composite n-back d' score; the CPT-IP d' and composite n-back d' scores were then standardized and averaged to yield the Objective Cognitive Score. Larger negative values reflect greater objective cognitive difficulties, whereas larger positive values indicate better objective cognitive performance. Fig. S2
