# Supplementary material for: Metacognitive discrepancies in schizotypy: Divergence between subjective and objective cognitive functioning
Source: Schizophr Res Cogn. 2026 Mar 18;45:100428. doi: 10.1016/j.scog.2026.100428 (PMC13019055; doi:10.1016/j.scog.2026.100428)
Supplement: Fig. S1 — Relationships between schizotypy dimensions and the Subjective Cognitive Score. Scatterplots illustrate associations between the Subjective Cognitive Score and (A) Cognitive-Perceptual, (B) Interpersonal, and (C) Disorganized subscales of the SPQ-BR, with fitted regression lines. The Subjective Cognitive Score was calculated by reversing and standardizing the WHODAS 2.0 Cognition domain score; higher values reflect better perceived cognitive functioning, and lower values reflect greater self-reported cognitive difficulties. [file mmc1.docx]

Panel A: Scatterplot of Cognitive-Perceptual schizotypy scores (x-axis) and the Subjective Cognitive Score (y-axis), with individual data points and a fitted regression line illustrating the negative association (higher Cognitive-Perceptual scores associated with lower subjective cognition).


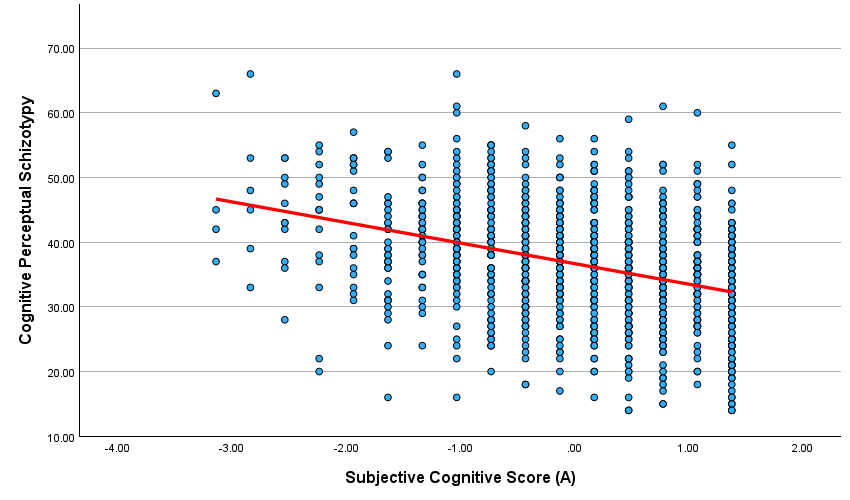


Panel B: Scatterplot of Interpersonal schizotypy scores (x-axis) and the Subjective Cognitive Score (y-axis), with data points and a fitted regression line showing a stronger negative association.


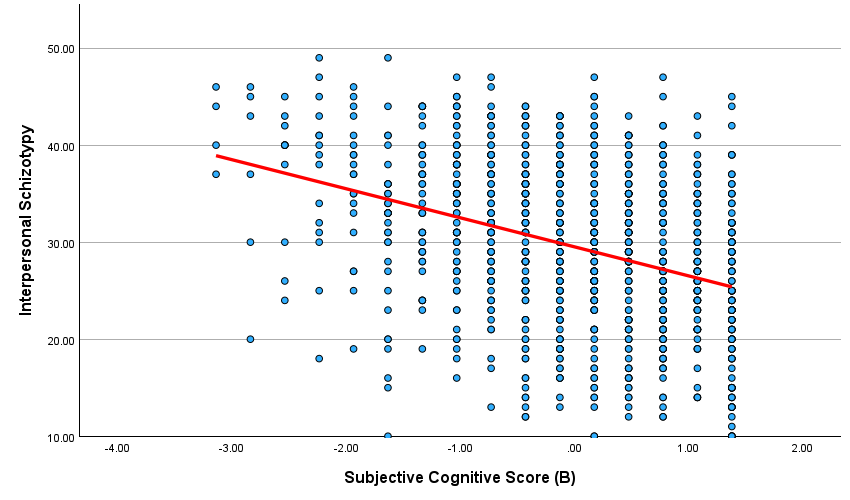


Panel C: Scatterplot of Disorganized schizotypy scores (x-axis) and the Subjective Cognitive Score (y-axis), with data points and a fitted regression line showing the strongest negative association.

**
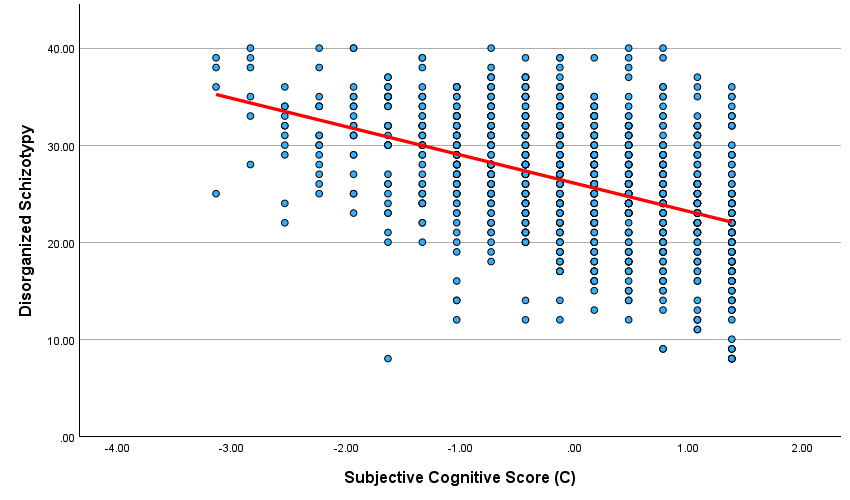
**

**Figure S1**
*Relationships between schizotypy dimensions and the Subjective Cognitive Score. Scatterplots illustrate associations between the Subjective Cognitive Score and (A) Cognitive-Perceptual, (B) Interpersonal, and (C) Disorganized subscales of the SPQ-BR, with fitted regression lines. The Subjective Cognitive Score was calculated by reversing and standardizing the WHODAS 2.0 Cognition domain score; higher values reflect better perceived cognitive functioning, and lower values reflect greater self-reported cognitive difficulties.*

*​*
