# Supplementary material for: Metacognitive discrepancies in schizotypy: Divergence between subjective and objective cognitive functioning
Source: Schizophr Res Cogn. 2026 Mar 18;45:100428. doi: 10.1016/j.scog.2026.100428 (PMC13019055; doi:10.1016/j.scog.2026.100428)
Supplement: Fig. S2 — Relationships between schizotypy dimensions and the Objective Cognitive Score. Scatterplots illustrate associations between the Objective Cognitive Score and (A) Cognitive-Perceptual, (B) Interpersonal, and (C) Disorganized subscales of the SPQ-BR, with fitted regression lines. The Objective Cognitive Score was calculated in multiple steps: a d' value was first computed for each participant on the CPT-IP, 2-back, and 3-back tasks; 2-back and 3-back d' values were standardized and averaged to create a composite n-back d' score; the CPT-IP d' and composite n-back d' scores were then standardized and averaged to yield the Objective Cognitive Score. Larger negative values reflect greater objective cognitive difficulties, whereas larger positive values indicate better objective cognitive performance. [file mmc2.docx]

Panel A: Scatterplot of Cognitive-Perceptual schizotypy scores (x-axis) and the Objective Cognitive Score (y-axis), with individual data points and a fitted regression line showing a negative association (higher Cognitive-Perceptual scores associated with lower objective performance).


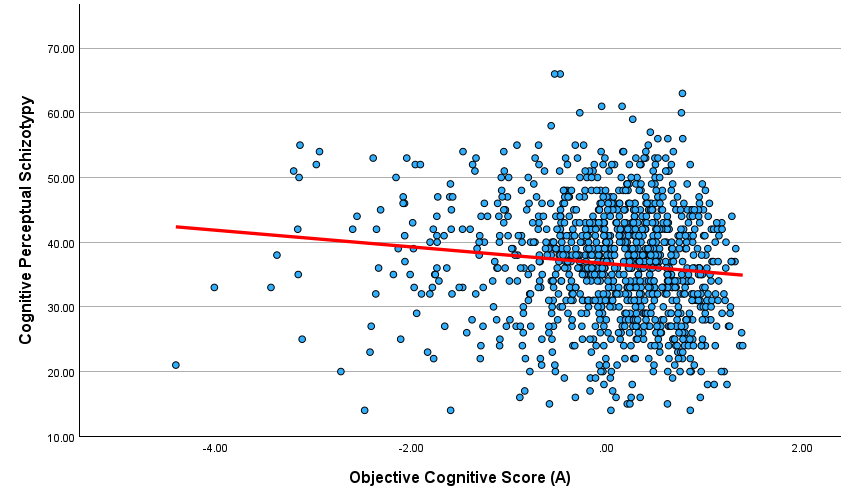


Panel B: Scatterplot of Interpersonal schizotypy scores (x-axis) and the Objective Cognitive Score (y-axis), with data points and a fitted regression line illustrating the nonsignificant association.


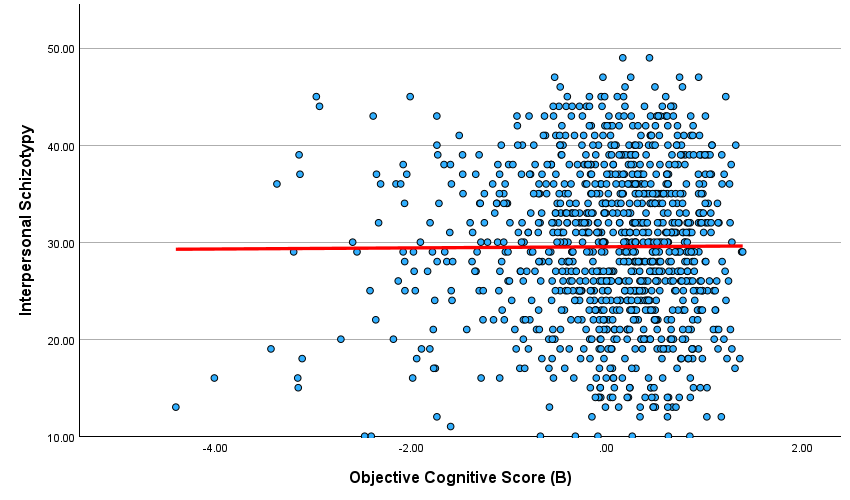


Panel C: Scatterplot of Disorganized schizotypy scores (x-axis) and the Objective Cognitive Score (y-axis), with data points and a fitted regression line showing a small positive association (higher Disorganized scores associated with slightly higher objective performance).


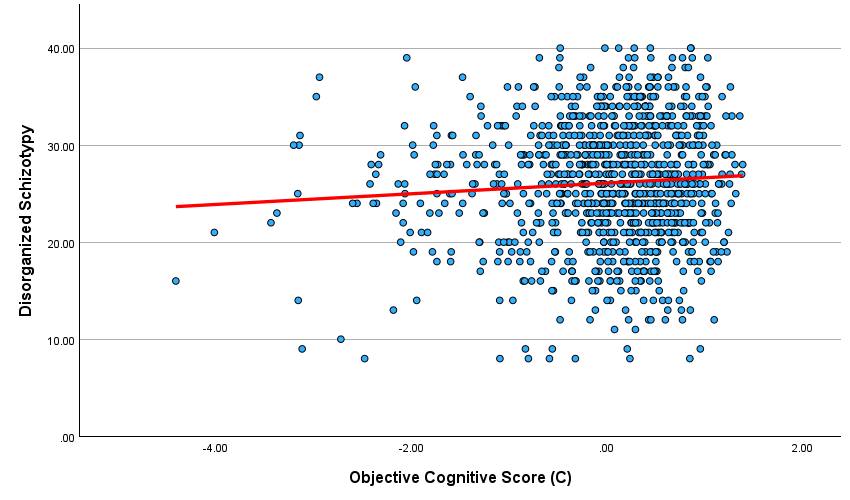


**Figure S2**
*Relationships between schizotypy dimensions and the Objective Cognitive Score. Scatterplots illustrate associations between the Objective Cognitive Score and (A) Cognitive-Perceptual, (B) Interpersonal, and (C) Disorganized subscales of the SPQ-BR, with fitted regression lines. The Objective Cognitive Score was calculated in multiple steps: a d′ value was first computed for each participant on the CPT-IP, 2-back, and 3-back tasks; 2-back and 3-back d′ values were standardized and averaged to create a composite n-back d′ score; the CPT-IP d′ and composite n-back d′ scores were then standardized and averaged to yield the Objective Cognitive Score. Larger negative values reflect greater objective cognitive difficulties, whereas larger positive values indicate better objective cognitive performance.*
